# Photosynthetic efficiency of perennial ryegrass (*Lolium perenne* L.) seedlings in response to Ni and Cd stress

**DOI:** 10.1038/s41598-023-32324-x

**Published:** 2023-04-01

**Authors:** Piotr Dąbrowski, Anna Jadwiga Keutgen, Norbert Keutgen, Edyta Sierka, Aneta Helena Baczewska-Dąbrowska, Jacek Mojski, Bogumiła Pawluśkiewicz, Leszek Sieczko, Hazem M. Kalaji

**Affiliations:** 1 Institute of Environmental Engineering, Warsaw University of Life Sciences – SGGW, Warsaw, Poland; 2 Department of Crop Sciences, Institute of Vegetables and Ornamentals, University of Natural Resources and Life Sciences, Vienna, Gregor-Mendel-Str. 33, 1180 Vienna, Austria; 3 Institute of Biology, Biotechnology and Environmental Protection, Faculty of Natural Sciences, University of Silesia in Katowice, 28 Jagiellonska, 40-032 Katowice, Poland; 4 Center for Biological Diversity Conservation in Powsin, Polish Academy of Sciences Botanical Garden, Warsaw, Poland; 5 Twój Swiat Jacek Mojski, Okrzei 39, 21-400 Lukow, Poland; 6 Fundacja Zielona Infrastruktura, Wiatraki 3E, 21-400 Lukow, Poland; 7 Department of Biometry, Institute of Agriculture, Warsaw University of Life Sciences—SGGW, Warsaw, Poland; 8 Institute of Technology and Life Sciences - National Research Institute, Falenty, Al. Hrabska 3, 05-090 Raszyn, Poland; 9 Department of Plant Physiology, Institute of Biology, Warsaw University of Life Sciences—SGGW, Warsaw, Poland

**Keywords:** Physiology, Plant sciences, Environmental sciences

## Abstract

Perennial ryegrass is a grass species used to establish lawns in urban areas where pollution is a major environmental problems. Cadmium (Cd) and nickel (Ni) contribute significantly to these pollutants and may cause photosynthetic limitation. The main objective of this work was to perform a comprehensive analysis of photosynthetic efficiency of perennial ryegrass seedlings under Cd and Ni stress. Some of the main indices of photosynthetic efficiency (prompt and delayed chlorophyll-a fluorescence signals and modulated reflectance at 820 nm) were compared with growth parameters. Two cultivars were tested: 'Niga' and 'Nira'. A decrease in photosystem (PS) II and PSI activity was observed. This was due to an increase in nonradiative dissipation of the PSII antenna, a decrease in PSII antenna size, or a decrease in the number of photosynthetic complexes with fully closed PSII RCs. Efficiency of electron transport was decreased. The effect on the modulated reflectance signal could indicate a restriction in electron flow from PSII to PSI. The correlation between photosynthetic efficiency parameters, such as Area, Fo, Fm, and Fv, and growth parameters, confirmed that some photosynthetic efficiency parameters can be used as indicators for early detection of heavy metal effects.

## Introduction

Heavy metal pollution has become a major problem worldwide^1^. High concentrations of heavy metals may have natural causes. The soils naturally containing heavy metals are found in various places around the world where metal ores come to the surface and decay through weathering. However, these sites are usually not considered relevant to human activities, so no attempts are made to detoxify them. Heavy metal contamination may also occur at high concentrations as a result of numerous human activities. Anthropogenic contamination may have various causes and can be observed in many countries around the world. The most obvious cause of anthropogenic heavy metal contamination of the environment is the activity of mining or refining industries, where emissions of dust particles and leakage of contaminated water from landfills or storage facilities are the main causes of environmental contamination^2–4^. In addition, road transport is an important source of metal pollution in heavily industrialized countries^5,6^.

Among the heavy metals that cause major environmental problems, important are cadmium and nickel. This is due to their high mobility in the environment and significant effects on humans and all biota. Cadmium has been reported to have the strongest toxic effect on wheat growth, followed by the metals Cu > Ni > Zn > Pb > Cr^7^. In other studies, cadmium was found to be the second most harmful heavy metal (after Hg) for root elongation in species such as *Triticum aestivum* L. and *Cucumis sativus* L^8^. Both elements are known to be available to plants from the air and soil^9^. Consequently, plants could be negatively affected if they take up these metals through leaves and roots. It should also be noted that cadmium is not an essential microelement for plant growth and development. In contrast, nickel is an essential nutrient for plants. However, the amount of Ni required for normal plant growth is very low^8^. Due to the increasing Ni pollution in the environment, it is also crucial to understand the functional role and toxic effects of Ni in plants.

The toxic effects of both metals on crucial physiological processes in plants, such as photosynthesis, water balance, mineral supply, and dark respiration, are generally comparable. For example, both compounds have been shown to cause similar disruption of photosynthesis at different structural–functional levels: Pigments and light uptake, thylakoid ultrastructure and photosynthetic electron transport, stomatal conductance and CO_2_ access, activities of Calvin cycle enzymes, etc^10–13^.

Photosynthesis is one of the most important processes in plants and is extremely sensitive to the changes in the environment. Its analysis is one of the most reliable techniques for estimating the effects of unfavorable environmental factors on plant growth and development. Under stress conditions, several processes are activated to distribute the excess energy absorbed by chlorophyll, including fluctuations in fluorescence emission and increased heat production. The measurements of chlorophyll *a* fluorescence (ChFl) are commonly used to analyze the response of photosynthesis to stress under various factors in all photosynthetic organisms^14–16^. ChFl kinetics may provide valuable information about the functional and structural features of photosynthetic electron transport^17,18^. The fluorescence of a dark-adapted leaf increases from the initial values (Fo) to the maximum values (Fm) in the first second of illumination and is represented as a curve with several points (labeled O, K, J, I, P)^19,20^. For this purpose, a high-frequency recording of ChFl emitted from dark-adapted leaves must be performed using a fluorimeter. Fluorescence kinetics reflects the photochemical efficiency of the photosynthetic apparatus and provides valuable information about the functional and structural features of the components involved in photosynthetic electron transport, referred to as PSII^21^. Based on the fluorescence transients, commonly known as Kautsky curves, it is also possible to calculate parameters that can be associated with the energy fluxes of absorption of light energy (ABS), trapping of excitation energy (TR), and conversion of excitation energy to electron transport (ET) per reaction center (RC) on the measured area of the samples (CS).

All redox reactions of photosynthetic electron transport between PSII and PSI and all electron transfer reactions in the RCs of PSII (donor and acceptor side) are reversible. Electron accumulation in the transport chain between PSII and PSI leads to a retransfer of electrons and the risk of recombination in the PSII RCs. In this situation, re-excitation of RC occurs as well as reoccupation (by rapid energy transfer) of the excited chlorophyll state of the PSII antenna. The light emission from the newly populated excited chlorophyll molecules is delayed compared to prompt fluorescence (PF). It is referred to as delayed fluorescence (DF) and is emitted prior to the utilization of excitation energy in the primary photochemical reaction^22^. After quenching, light is emitted in the red-infrared spectrum and occurs for a short time after PF decay. DF is emitted only by PSII and decays as a polyphasic function in different time domains: Nanoseconds, microseconds, milliseconds, seconds and even minutes^22,23^. Similar to PF, DF emission may be described as a fluorescence transient. A characteristic feature of this curve is the presence of several maxima and minima. Their number and amplitude depend on the kinetic components of the measured DF. According to^22,23^ the maxima are denoted by I and the minima by D letters.

The modulated reflectance signal (MR) is measured at 820 nm and it provides information about electron transport adjacent to plastoquinone (PQ) and to PSI acceptors^24^, indicating the changes in the redox states of PSI RCs and plastocyanin (PC).

Perennial ryegrass (*Lolium perenne* L.) is one of the most widespread grass species in Europe. It is widely used as a forage crop and as an alternative and renewable bioenergy source. The turf cultivars of perennial ryegrass are widely used for the establishment of lawns in urban areas^25,26^. Urban green spaces have numerous functions. Maintained lawns increase the aesthetic value of the whole city and are involved in phytoremediation, which leads to improvement of air and soil quality^25^. On the one side, seedlings are particularly more responsive to different environmental conditions (e.g. water availability, photosynthetic photon flux density), and it is the development in this phase that has the greatest impact on the later functioning of the entire lawn^27^. On the second hand, despite that seedling are more responsive than mature plants, so far, there have been no studies on the influence of heavy metals on the photosynthetic efficiency of the *Lolium perenne* seedling. Numerous studies have demonstrated the possibility of simultaneously measuring PF, DF, and modulated reflectance to reveal changes in photosynthetic efficiency under different environmental factors, such as various abiotic and biotic stresses^28,29^. However, there is a lack of information on how cadmium and nickel contamination affect the seedlings of perennial ryegrass turfgrass cultivars. Literature is lacking on the ability of chlorophyll *a* fluorescence to detect changes in PSII and PSI in seedlings of this species. In this work, the multifunctional plant efficiency analyzer (MPEA-2; Hansatech Instruments, UK) was used to perform simultaneous measurements of PF, DF, and MR_820_ signals in seeds of two grass cultivars of perennial ryegrass exposed to cadmium- and nickel-induced stress. It was hypothesized that the stress could affect multiple sites of the photosynthetic electron transport chain in different ways depending on the heavy metal and cultivar. We focused on changes in the state of the photosynthetic apparatus in response to these two treatments to find possible marker parameters of these stresses.

The main objective of this study was to perform the detailed in vivo analysis and to compare of the changes in PSII photochemistry induced by Ni and Cd stress in seedlings of two turfgrass cultivars of perennial ryegrass by analyzing parameters derived from the prompt and delayed chlorophyll fluorescence recordings and from MR_820_ signal. In addition, we tested whether the chlorophyll fluorescence data could be used to discriminate cultivars with better resistance to soils contaminated with these metals. The results would be useful to understand the photosynthetic performance of perennial ryegrass under stress conditions and would increase our knowledge of the photosynthetic system under heavy metal contamination.

## Results

### The heavy metals concentration in leaves

Averaged concentration of Cd in ‘Niga’ cultivar grooving on substrate contaminated with 50 mg of this elements was 0.27 mg × kg^−1^ D.M., but in plants on substrate contaminated with 100 mg was 7.4 mg × kg^−1^ D.M (Table 1). Similar phenomena was noted in ‘Nira’ cultivar. The averaged concentration of Cd was 2.9 and 6.1 mg × kg^−1^ D.M. respectively.

**Table 1:** Cadmium and nickel concentration in leaves [mg × kg^−1^ D.M.] ± S.D of two perennial ryegrass turf cultivars (‘Niga’ and ‘Nira’) under different Ni and Cd treatment (0; 50 and 100 mg × 100 g^−1^ of substrate).

Cultivar	Treatment	Cadmium		Nickel	
		Mean	SD	Mean	SD
Niga	Control	0.0a	0.0	0.0	0.0
	50 mg Cd	2.7b	0.7	–	–
	100 mg Cd	7.4c	0.9	–	–
	50 mg Ni	–	–	23.3a	6.7
	100 mg Ni	–	–	37.3b	2.1
Nira	Control	0.0	0.0	0.0	0.0
	50 mg Cd	2.9a	0.7	–	–
	100 mg Cd	6.1b	1.3	–	–
	50 mg Ni	–	–	21.0a	9.2
	100 mg Ni	–	–	36.7b	3.2

Means within one cultivar marked by different letters represent statistically significant differences (*p* < 0.05, n = 6).

Averaged concentration of Ni in Niga cultivar growing on substrate contaminated with 50 mg of this elements was 23.3 mg × kg^−1^ D.M., and in plants grooving on substrate contaminated with 100 mg of Ni it was 37.3 mg × kg^−1^ D.M. The averaged concentration of Ni in Nira cultivar was 21.0 and 3.67 mg × kg^−1^ D.M. respectively.

### The chlorophyll and total nitrogen concentration in leaves

The impacts of both metals were reflected in modifications of the mean values of chlorophyll *a* and *b* concentration in leaves tissue. Averaged concentration of Chl *a* in ‘Niga’ cultivar growing on substrate not contaminated was 2.39 mg × g^−1^ F.M., and Chl *b* was 0.78 mg × g^−1^ F.M. (Table 2). It was noted, that values of both pigments in plants grooving on substrate contaminated with Cd and Ni decreased. Moreover, Ni caused deeper decreasing of this pigment than Cd.

**Table 2:** Chlorophyll and total nitrogen concentration in leaves [mg × g^−1^ F.W.] ± S.D of two perennial ryegrass turf cultivars (‘Niga’ and ‘Nira’) under different Ni and Cd treatment (0; 50 and 100 mg × 100 g^−1^ of substrate).

Cultivar	Treatment	Chl *a*		Chl *b*		Total nitrogen	
		Mean	SD	Mean	SD		
Niga	Control	2.39a	0.21	0.78a	0.06	21.20a	1.05
	50 mg Cd	2.01a	0.08	0.63ab	0.05	18.67a	1.20
	100 mg Cd	1.87b	0.11	0.62b	0.05	18.00a	2.15
	50 mg Ni	1.53bc	0.07	0.52b	0.04	18.17a	1.00
	100 mg Ni	1.19bc	0.09	0.39c	0.03	18.57a	0.87
Nira	Control	2.37a	0.30	0.85a	0.09	20.13a	1.44
	50 mg Cd	2.16ab	0.11	0.72ab	0.05	20.16a	1.95
	100 mg Cd	2.10b	0.05	0.70b	0.03	17.93a	1.11
	50 mg Ni	1.56b	0.09	0.54b	0.04	16.93a	0.89
	100 mg Ni	1.56b	0.06	0.52b	0.03	18.23a	0.92

Means within one cultivar marked by different letters represent statistically significant differences (*p* < 0.05, n = 6).

Averaged concentration of chlorophylls in ‘Niga’ cultivar growing on uncontaminated substrate was 2.37 and 0.30 mg × g^−1^ F.M., as in case of ‘Niga’ cultivar, in plants growing on substrate contaminated with Cd the changes were less pronounced that in plants crowing on substrate contaminated by Ni.

The average total nitrogen content in ‘Niga’ growing on uncontaminated substrate was 21.20 mg × g^−1^ F.M., and in ‘Nira’ cultivar it was 20.13 mg × g^−1^ F.M. There were no significant changes in values of this parameters in plants growing on contaminated substrate.

### Prompt chlorophyll *a* fluorescence

**Figure 1:**
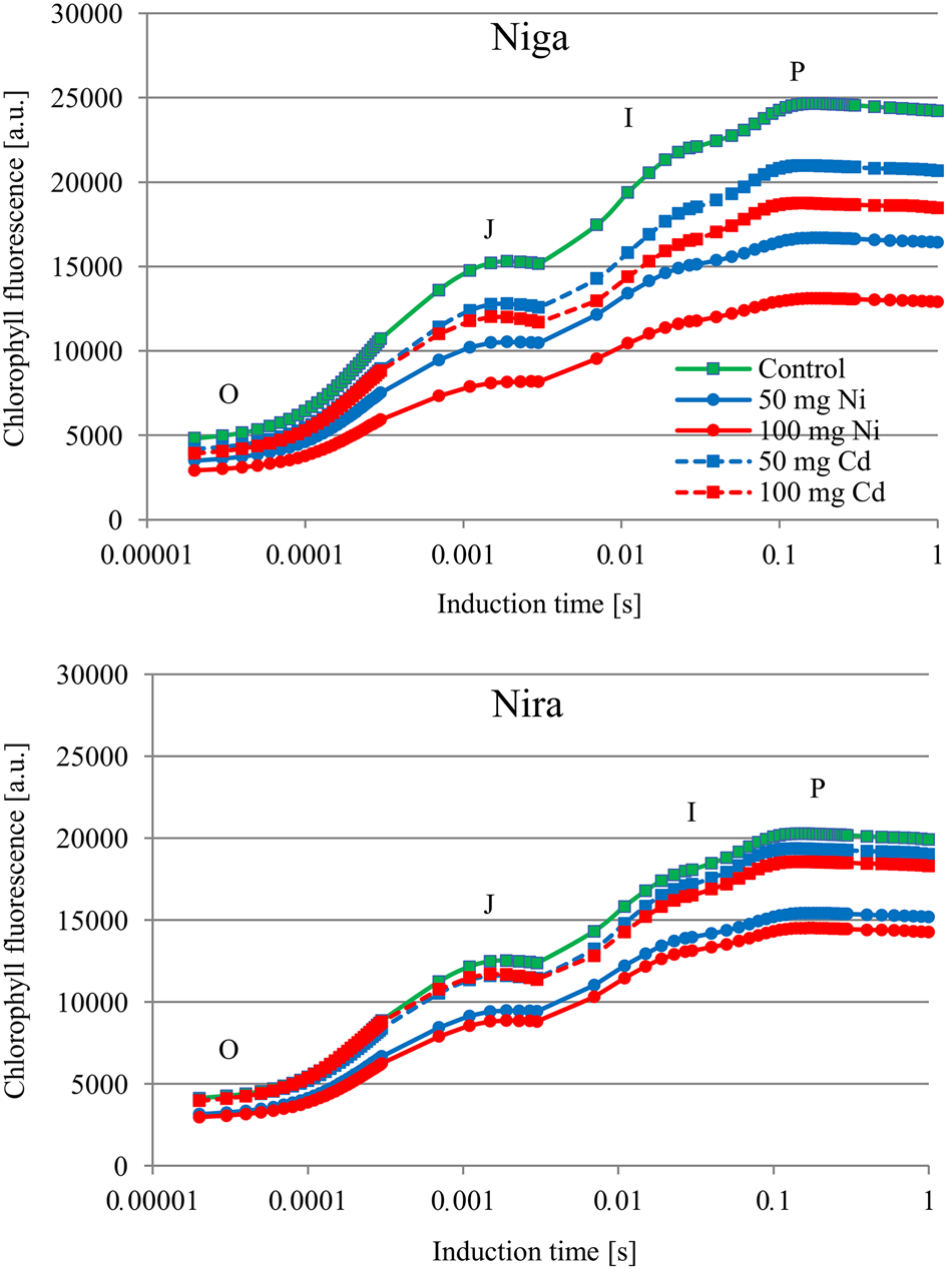
Induction curves of chlorophyll* a* fluorescence [relative units] of two perennial ryegrass turf cultivars (‘Niga’ and ‘Nira’) under different Ni and Cd treatment (0; 50 and 100 mg × 100 g^−1^ of substrate) (n = 6).

The two heavy metals Ni and Cd significantly affected the intensity of the fluorescence signal of perennial ryegrass seedlings. However, these changes were also dependent on cultivar and concentration (Fig. 1). In the case of cultivar ‘Niga’, the most pronounced changes were observed in plants treated with 100 mg Ni × 100 g^−1^ substrate, and the measured fluorescence signal was decreased at the J point compared to the control samples. In both treatments with Ni and in the case of the treatment with 100 mg Cd × 100 g^−1^ substrate, the changes occurred at the I point. In the case of the cultivar ‘Nira’, only nickel at both concentrations caused a significant decrease in the transient at the J-point.

**Figure 2:**
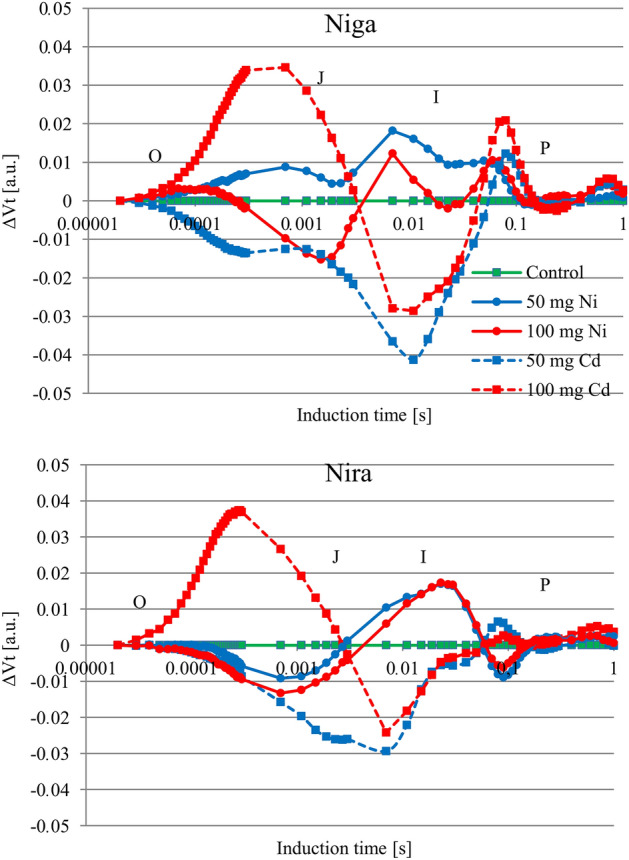
Differential curves of ΔV_t_ [relative units, obtained by subtracting control curve from the first sample] of two perennial ryegrass turf cultivars (‘Niga’ and ‘Nira’) under different Ni and Cd treatment (0; 50 and 100 mg × 100 g^−1^ of substrate) (n = 6).

The course of normalized OJIP fluorescence transients measured in stressed plants of both cultivars differed from those recorded in nonstressed plants (Fig. 2). The most important changes in the curve of stressed plants were observed in the I–P phase. In the O–J phase, the curve of plants treated with 100 mg Cd × 100 g^−1^ had a higher course than that of control plants. In the cultivar ‘Niga’ this phenomenon was also observed in the plants treated with 50 mg Cd × 100 g^−1^.

**Figure 3:**
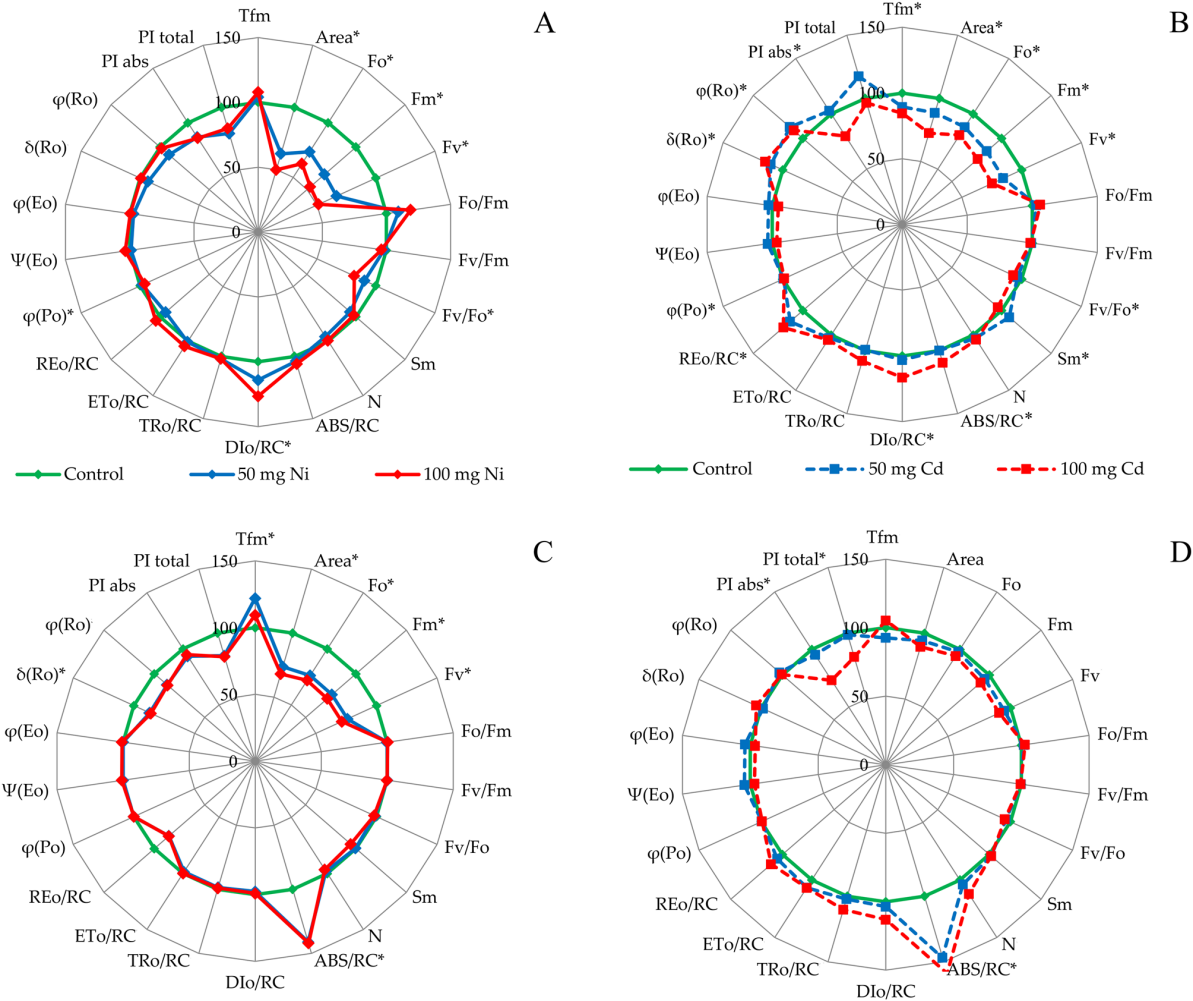
JIP-test parameters (relative units, normalized to the values from control conditions) as radar plots of two perennial ryegrass turf cultivars. (**A**) ‘Niga’ treated by Ni; (**B**) ‘Niga’ treated by Cd; (**C**) ‘Nira’ treated by Ni; (**D**): ‘Nira’ treated by Cd. The concentration of Ni and Cd were: 0; 50 and 100 mg × 100 g^−1^ of substrate. Means marked by different letters represent statistically significant differences (*p* < 0.05, n = 6) delayed chlorophyll *a* fluorescence.

The impacts of both metals were reflected in modifications of the mean values of some parameters deduced from the JIP measurements. However, this interaction was largely dependent on the cultivar and the metal itself (Fig. 3). In both cultivars, Ni caused a decrease in the parameter Area, F_o_, F_m_, F_v_. Only in the ‘Niga’ cultivar this metal caused an increase in DI_o_/RC. In the ‘Nira’ variant, the value of the ABS/RC parameter increased, but the δ_Ro_ and φ_Ro_ values decreased. Cd reduced the parameters F_o_, F_m_, F_v_, PI_abs_ and PI_total_ in both cultivars. ‘Niga’ showed an increase in δ_Ro_ and φ_Ro_ values, which was not found in ‘Nira’. In this cultivar, however, an increase in values ABS/RC parameter was found.

**Figure 4:**
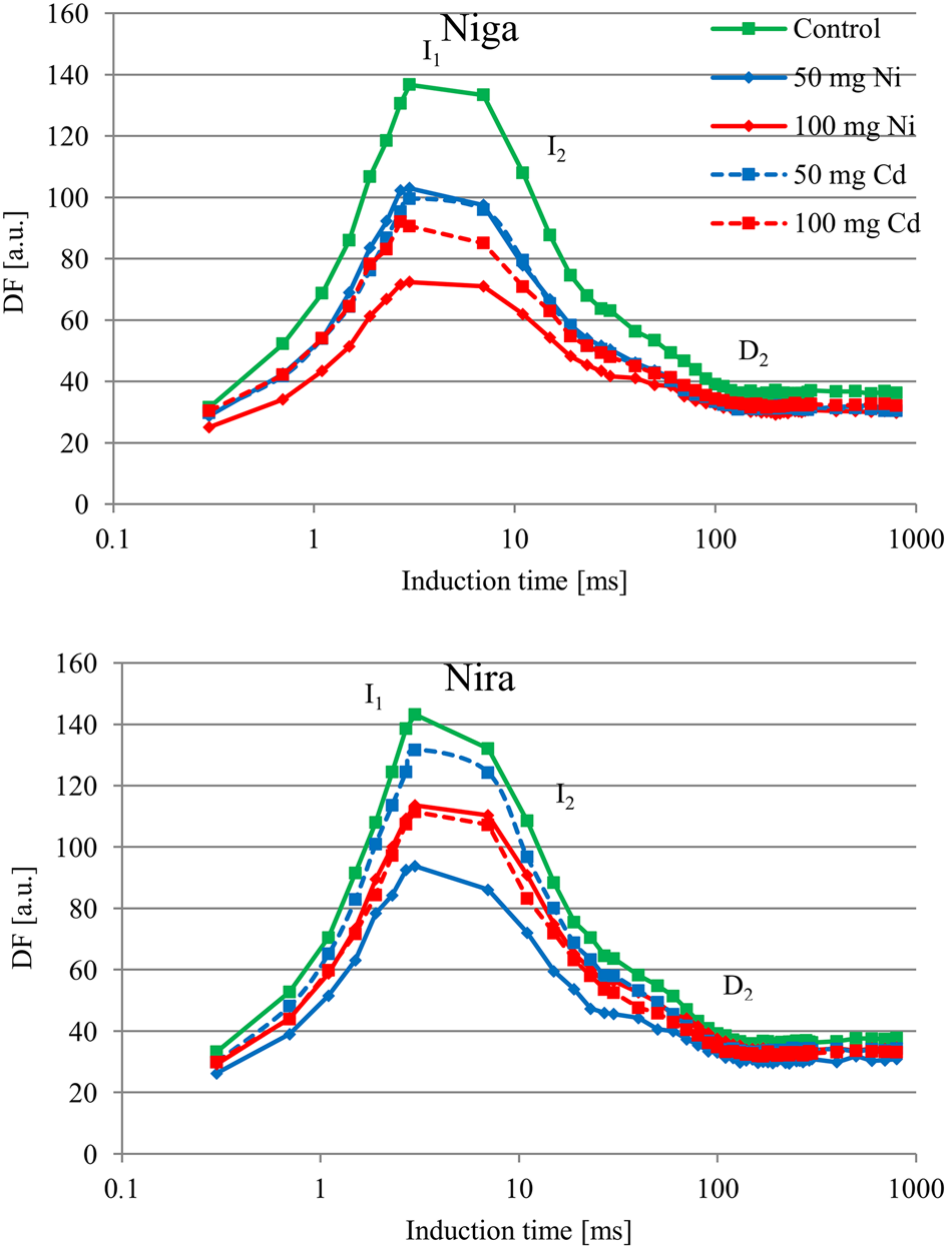
Delayed fluorescence induction curves [relative units] of two perennial ryegrass turf cultivars (‘Niga’ and ‘Nira’) under different Ni and Cd treatment (0; 50 and 100 mg × 100 g^−1^ of substrate) (n = 6).

In this work we focused on the first recorded delayed fluorescence (DF) decay component that appeared within a time range of a microsecond. In this part of DF induction kinetics (which is initial, during the first 1 s of illumination) the emission was of principal share. The intensity of the DF signal was reduced by both heavy metals. However, the degree of their impact varied with cultivar and metal (Fig. 4). For the ‘Niga’ cultivar, Ni and Cd in concertation of 50 mg × 100 g^−1^ caused a comparable decrease in the intensity of curves. The first maximum of curve (I_1_) decreased from 138.7 rel. u. to 100.7 and 104.5 rel. u., respectively (Table 3). In this cultivar treating with the concentration of 100 mg × 100 g^−1^ caused a different reaction depending on the element. Cd in this concentration caused decreasing of the I_1_-point to 94.4 rel. u., but Ni in this concertation caused decreasing to 75.3 rel. u.

**Table 3:** (I_1 _− D_2_)/D_2_ and I_1_/I_2_ ratios ± S.D. [relative units] of two perennial ryegrass turf cultivars (‘Niga’ and ‘Nira’) under different Ni and Cd treatment (0; 50 and 100 mg × 100 g^−1^ of substrate).

Cultivar	Treatment	I_1_		I_2_		D_2_		I_1_/I_2_		(I_1 _− D_2_)/D_2_	
		Mean	SD	Mean	SD	Mean	SD	Mean	SD	Mean	SD
Niga	Control	138.7a	12.8	62.9a	4.0	34.1a	2.1	2.2a	0.1	3.1a	0.3
	50 mg Cd	100.7b	9.8	48.9b	4.2	29.4b	0.5	2.0ab	0.1	2.4b	0.3
	100 mg Cd	94.4bc	17.2	48.0b	5.3	30.2b	1.9	1.9ab	0.2	2.1b	0.5
	50 mg Ni	104.5b	31.1	50.3b	8.1	29.8b	3.4	2.0ab	0.4	2.4b	0.8
	100 mg Ni	75.3b	17.2	41.7b	5.5	28.2b	2.9	1.8b	0.2	1.6c	0.4
Nira	Control	144.2a	16.5	63.6a	7.5	34.1a	3.1	2.3a	0.3	3.2a	0.5
	50 mg Cd	132.4b	20.5	58.1b	5.2	31.4a	1.5	2.2a	0.1	3.2ab	0.4
	100 mg Cd	113.2b	22.1	52.6b	7.2	30.8a	1.9	2.1a	0.2	2.6ab	0.6
	50 mg Ni	94.8c	47.6	45.5c	15.0	28.4a	5.2	1.9a	0.5	2.2b	1.3
	100 mg Ni	114.7b	17.0	56.2b	6.2	31.5a	1.5	2.0a	0.1	2.6ab	0.4

Means within one cultivar marked by different letters represent significant differences (*p* < 0.05, n = 6).

Cadmium (concertation of 50 mg × 100 g^−1^) did not cause any significant changes in the ‘Nira’ cultivar. Significant changes of the courses of the curves were noted after the application of 100 mg × 100 g^−1^ of both elements. In these treatments the I_1_ parameter decreased to 113.7 and 114.7 rel. u. Similar changes were measured after application of 50 mg × 100 g^−1^ Ni. The value of the I_1_ parameter decreased to 94.4 rel. u. It should be noted that both metals caused significant changes in the I_2_ and D_2_ parameters and in the I_1_/I_2_ and (I_1_–D_2_)/D_2_ ratios.

### Modulated light reflection signal measured at 820 nm

**Figure 5:**
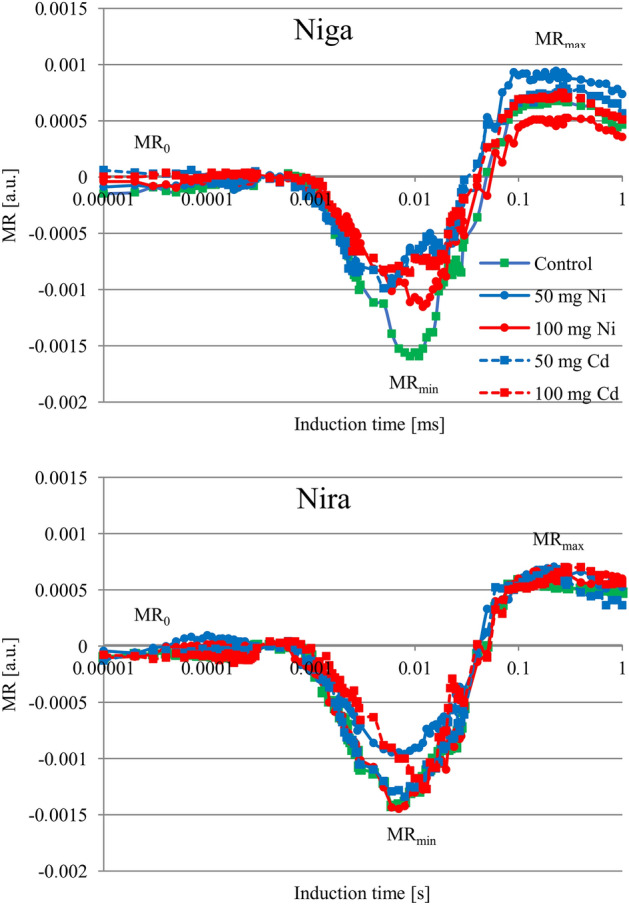
Kinetics of modulated light reflection at 820 nm [relative units, all data are normalized to the initial measured value of the signal] of two perennial ryegrass turf cultivars (‘Niga’ and ‘Nira’) under different Ni and Cd treatment (0; 50 and 100 mg × 100 g^−1^ of substrate) (n = 6).

The reflectance at 820 nm was significantly affected by stress application in both cultivars (Fig. 5, Table 4). In the 'Niga' cultivar, MR_min_ increased due to both metals, regardless of their concertation. The most significant increase of this parameter was noted in ‘Nira’ treated by 50 mg Ni × 100 g^−1^. The addition of nickel of 100 mg slightly modified the shape of the curves at this point, however this was not statistically significant. Other treatments caused similar courses of curves as in control plants. Based on the statistical analysis, it can be concluded that in the ‘Niga’ cultivar the relative rates of P_700_ oxidation (ΔMR_fast_) parameter decreased significantly in comparison to control in all plants treated with both metals. In the ‘Nira’ cultivar only 50 mg Ni × 100 g^−1^ caused a significant reduction of this parameter. P_700_^+^ re-reduction (ΔMR_slow_) in the ‘Niga’ cultivar was significant decreased by all treatments except 50 mg Cd × 100 g^−1^. However, in the ‘Nira’ cultivar only 50 mg Ni × 100 g^−1^ caused significant differences in the comparison with the control.

**Table 4:** Relative rates of P_700_ oxidation (ΔMR_fast_) and P_700_^+^ re-reduction (ΔMR_slow_) ± S.D. [relative units] calculated from the MR_820_ signal of two perennial ryegrass turf cultivars (‘Niga’ and ‘Nira’) under different Ni and Cd treatment (0; 50 and 100 mg × 100 g^−1^ of substrate).

Cultivar	Treatment	ΔMR_fast_		ΔMR_slow_	
		Mean	SD	Mean	SD
Niga	Control	25.3a	10.0	32.9a	12.6
	50 mg Cd	17.6b	5.0	29.9a	6.1
	100 mg Cd	14.5c	4.2	24.6a	3.4
	50 mg Ni	13.6c	6.9	23.6a	5.4
	100 mg Ni	16.1b	2.2	20.9a	4.2
Nira	Control	21.9a	4.8	30.5a	5.4
	50 mg Cd	22.2a	7.0	30.5a	5.8
	100 mg Cd	20.3a	3.8	28.3a	4.9
	50 mg Ni	13.3b	2.8	21.4a	4.8
	100 mg Ni	20.4a	5.3	30.0a	6.4

Means within one cultivar marked by different letters represent significant differences (*p* < 0.05, n = 6).

### Growth parameters

**Figure 6:**
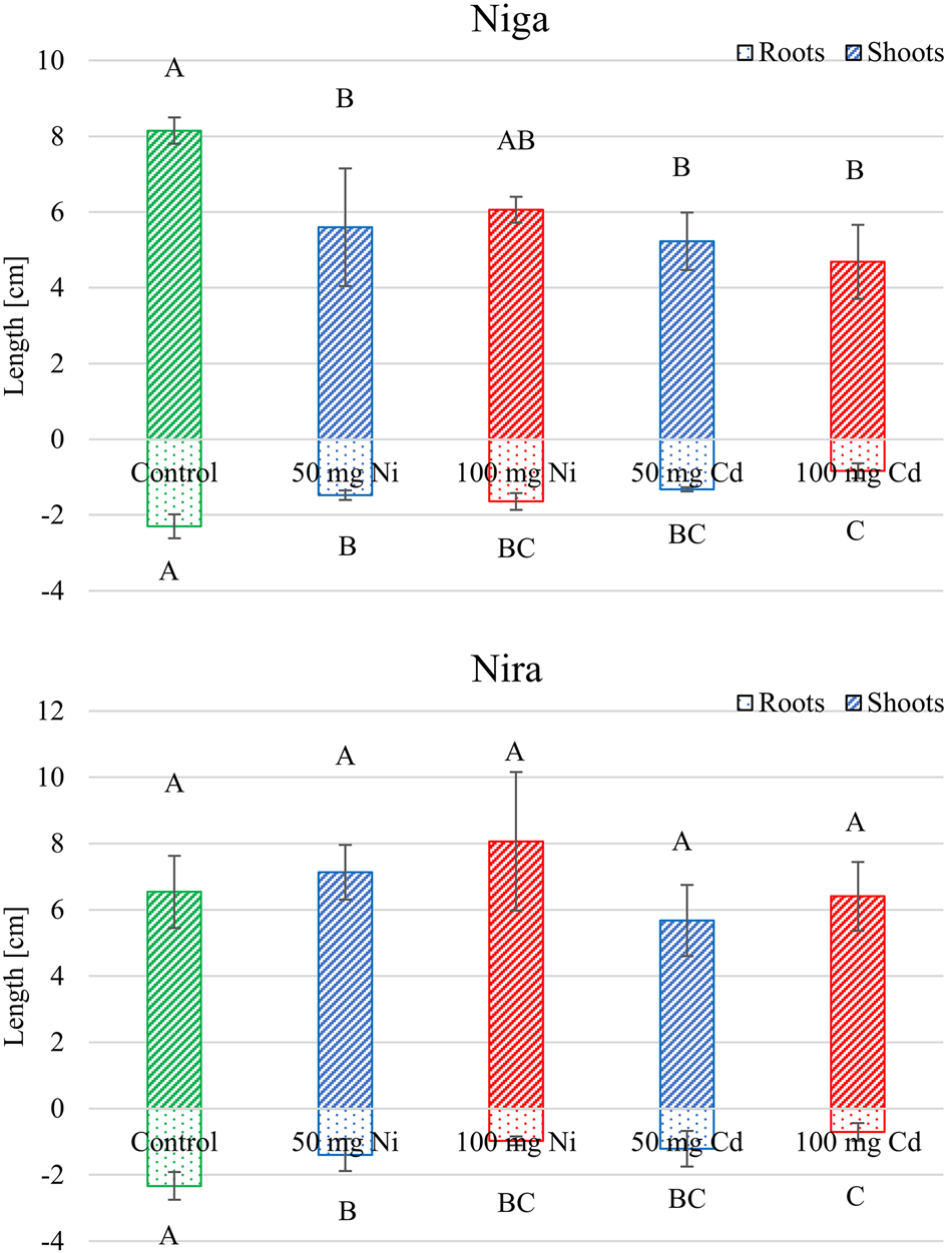
Length of shoots and roots of two perennial ryegrass turf cultivars (‘Niga’ and ‘Nira’) under different Ni and Cd treatment (0; 50 and 100 mg × 100 g^−1^ of substrate). Means within one cultivar marked by different letters represent significant differences (*p* < 0.05, n = 6).

Averaged length of ‘Niga’ shoots in control conditions was 8.2 cm (Fig. 6). Both metals caused significant decreasing of this parameter, beside 100 mg of Ni. Length of roots also was influenced by these stressors. Average value decreased from 2.3 to 0.8–1.6 cm. In ‘Nira’ cultivar there were no influence of any metals on length of shoots. However, both metals caused decreasing of length of roots. This parameter was reduced from 2.4 to 0.8 cm by 100 mg of Cd. Others treatments also caused significant reduction. 50 mg of Cd caused reduction to 1.2 cm, and Ni caused reduction to 1.4 and 1.0 cm respectively.

### Relationships between photosynthetic efficiency and growth parameters

On the basis of the statistical analysis significant correlations between plant photosynthetic efficiency parameters and plant growth parameters have been noticed (Table 5). However, these correlations were cultivar and stressor dependent. In the ‘Niga’ cultivar treated with cadmium, most of the parameters of photosynthetic efficiency were correlated with the length of the shoots and the length of the roots. In this variety treated with nickel, a high correlation was found between the parameters describing the curve (such as F_o_, F_m_, Area) and the length of both organs. However, there was no relationship between plant growth and DF and MR820.

**Table 5:** Correlation coefficients (r) between photosynthetic efficiency parameters obtained from prompt (JIP-test), delayed fluorescence (I_1_; I_2_; D_2_; I_1_/I_2_; (I_1 _− D_2_/D_2_)), and phases of reflection intensity (ΔMR_fast_; ΔMR_slow_) and root and shoot length (correlations significant at *p* < 0.05, n = 6).

Parameter	Niga				Nira			
	Cd		Ni		Cd		Ni	
	Roots	Shoots	Roots	Roots	Roots	Shoots	Roots	Shoots
Chl *a*	0.89	0.68	0.81	0.61	0.38	0.09	0.71	0.72
Chl *b*	0.84	0.64	0.77	0.57	0.57	0.04	− 0.27	− 0.31
Chl *a* + *b*	0.89	0.67	0.79	0.60	0.44	0.08	0.72	0.72
Nitrogen	0.45	0.47	0.66	0.45	0.13	− 0.45	− 0.27	− 0.31
Tf_m_	0.90*	0.77*	− 0.03	− 0.38	0.00	0.33	0.48	0.06
Area	0.81*	0.74*	0.78*	0.65*	0.28	0.02	0.77*	0.60
F_o_	0.77*	0.74*	0.80*	0.64*	0.16	− 0.15	0.74*	0.61
F_m_	0.84*	0.79*	0.79*	0.66*	0.36	0.07	0.79*	0.52
F_v_	0.85*	0.80*	0.79*	0.67*	0.38	0.10	0.78*	− 0.50
F_o_/F_m_	− 0.86*	− 0.75*	− 0.63*	− 0.64*	− 0.39	− 0.28	− 0.30	− 0.03
F_v_/F_m_	0.86*	0.74*	0.63	0.64	0.39	0.29	0.30	0.04
F_v_/F_o_	0.87*	0.75*	0.65	0.64	0.49	0.30	0.39	− 0.01
Sm	0.10	0.01	0.27	0.14	− 0.44	− 0.25	0.16	− 0.52
N	− 0.40	− 0.19	0.00	− 0.13	− 0.78*	− 0.12	0.04	− 0.55
ABS/RC	− 0.71*	− 0.36	− 0.59	− 0.58	− 0.72*	− 0.46	− 0.66	0.01
DI_o_/RC	− 0.80*	− 0.53	− 0.64	− 0.65	− 0.60	− 0.19	− 0.28	− 0.08
TR_o_/RC	− 0.67	− 0.30	− 0.49	− 0.49	− 0.74*	0.05	− 0.18	− 0.20
ET_o_/RC	− 0.57	− 0.63	− 0.49	− 0.26	− 0.81*	− 0.08	− 0.33	0.00
RE_o_/RC	− 0.80*	− 0.54	− 0.19	0.01	− 0.58	0.12	0.31	− 0.32
φ_Po_	0.86*	0.75*	0.63	0.64	0.39	0.28	0.30	0.03
Ψ_Eo_	0.32	− 0.12	− 0.16	0.09	− 0.12	− 0.16	− 0.31	0.15
φ_Eo_	0.43	− 0.01	0.16	0.40	− 0.01	− 0.09	− 0.23	0.22
δ_Ro_	− 0.81*	− 0.45	0.15	0.26	− 0.31	0.23	0.49	− 0.38
φ_Ro_	− 0.63	− 0.51	0.21	0.42	− 0.28	0.17	0.41	− 0.32
PI_ABS_	0.66	0.26	0.49	0.62	0.57	− 0.03	0.28	− 0.02
PI_total_	− 0.03	− 0.17	0.52	0.67*	0.46	0.12	0.66	− 0.33
I_1_	0.87*	0.58	0.61	0.62	0.38	0.07	0.71*	− 0.06
I_2_	0.87*	0.56	0.66	0.58	0.38	0.17	0.48	− 0.02
D_2_	0.83*	0.75*	0.73	0.62	0.39	0.23	0.45	− 0.17
I_1_/I_2_	0.80*	0.52	0.46	0.68*	0.24	− 0.07	0.78*	− 0.04
I_1 _− D_2_/D_2_	0.79*	0.41	0.50	0.59	0.28	− 0.02	0.71*	0.04
ΔMR_fast_	0.65*	0.65	0.48	0.72*	0.02	0.52	0.45	− 0.15
ΔMR_slow_	0.42	0.56	0.36	0.62	0.05	0.41	0.36	− 0.13

In the ‘Nira’ cultivar, much less dependence was found, and only between the individual parameters of photosynthesis efficiency and the length of the roots. In plants of this cultivar treated with cadmium, these parameters were N, ABS/RC, Tr_o_/RC and ET_o_/RC. In nickel-treated plants these parameters were: Area, F_o_, F_m_, F_v_, I1, I_1_/I_2_, I_1_-D_2_/D_2_.

**Figure 7:**
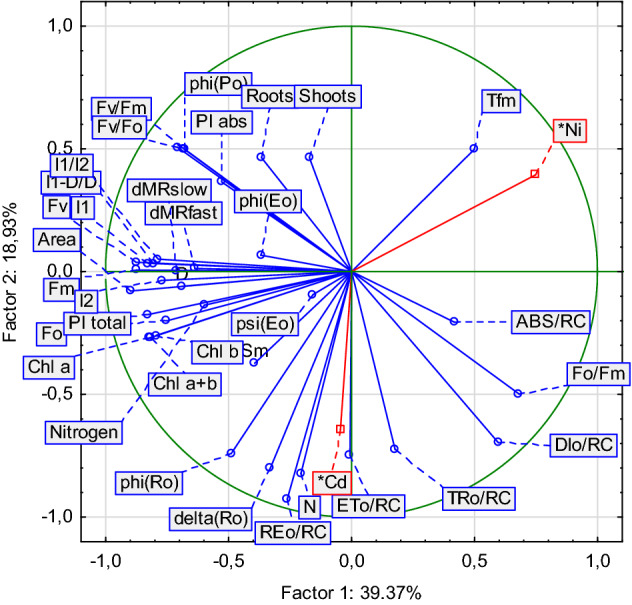
Principal component analysis of two heavy metals (Ni and Cd) effects on the photosynthetic machinery of leaves of two species of ryegrass: ‘Niga’; ‘Nira’.

A multiparametric analysis was used to evaluate the stress effects of both heavy metals in perennial ryegrass in order to identify parameters that are most sensitive to the plant stress response. In the vector graphs (Fig. 7) the relative “contribution” of each input variable to the formation of the principal components (Factor 1 and Factor 2) is presented. The magnitude of the vector is an indicator of the stressor influence on corresponding parameter and the vector direction depends on this impact on the Factor 1 and Factor 2 values.

The modifications in the first principal component (Factor 1) determined 39.37% of total changes, the second component (Fator 2) reflected 18.93%. Based on this analysis it can be concluded that both varieties has similar response to stress. However, Cd and Ni caused different response of photosynthetic apparatus of both varieties. The analyzed parameters of the JIP-test, DF and phases of reflection intensity had different sensitivity to stresses, and contributed differently to the calculation of the principal components. The parameters can be merged in the few groups. Independent group was formed by the DF parameters and ΔMR_fast_ and ΔMR_slow_, which were merged with Area, F_o,_ F_m_, F_v_, PI_total._ Second group consist F_v_ /F_m_, F_v_ /F_o_, φ_Eo_, φ_Po,_ Ψ_Eo_, PI_abs._ The third group consist of F_o_/F_m_ TR_o_/RC, ABS/RC, and DI_o_/RC. It should be noted, that length of shoots and length of roots also were merged to one group, both of them corelated with F_v_/F_m_, PI_ABS_ and φ_Ro._ The factor analysis used allowed to reduce the 28-dimensional space (the number of variables used in the PCA) to an easier-to-interpret space of 2 dimensions. Since factors 1 and 2 explain more than 60% of the overall variability, it is possible to identify the most important groups of parameters correlated with the root and shoot.

## Discussion

The tolerance and physiological status of plants can be evaluated using various methods of biochemistry, ecology, plant physiology, genetics, and biophysics. In this study, measurement of chlorophyll *a* fluorescence was proposed as a suitable and sensitive bioindicator of plant stress in response to heavy metal contamination. It allows obtaining large amounts of detailed data on each step of the photosynthetic process. It is also very sensitive to changes in the physiological state of a plant in vivo^29–31^. To describe the response of the photosynthetic apparatus of perennial ryegrass seedlings to Cd and Ni exposure, the simultaneous measurements of PF, DF and MR_820_ were performed. This allows the analysis of changes in important energetic properties of PSII and PSI as well as stress-induced processes in the light phase of photosynthesis.

The harmful effects of heavy metals depend on their intake. In general, Ni is absorbed relatively more compared to Cd^32^. Inhibition of chlorophyll synthesis is one of the main reasons for lower photosynthetic activity^33^. However, the symptoms of metal toxicity, shown by the reduction of biomass, pigment quantity, and photosynthetic activity, depend not only on the metal but also on the variety tested^34^. The negative influence on photosynthetic properties seems to be a main reason for the reduced growth and development of plants^34^. These authors suggest that such a decrease in photosynthetic activity may be the result of a reduction in chlorophyll content caused by the heavy metals. Here, evidence is presented that the sensitivity of the photosynthetic system to different metal concentrations may be coupled to the cultivar. According to Khalid et al.^35^, the reduction in photosynthetic rate is a clear consequence of heavy metal toxicity. Our results for both ryegrass cultivars agree with data from other researchers who found reduced photosynthetic activity in soybean^36^, brassica^37^, maize^38^, wheat^39^, spinach, and fenugreek^40^ under both metal stresses. However, these experiments were conducted on fully developed plants. In our study, it was demonstrated for the first time that the extent of photosynthesis reduction depends on the cultivar even in young turfgrass cultivars. This is of crucial importance for the selection of plants for the regeneration of degraded soils. In addition, Aqeel^13^ found that Cd concentration was higher in roots than in shoots. In contrast, Ni concentration was higher in shoots than in roots. This could explain the fact that photosynthesis in perennial ryegrass is more strongly influenced by Ni.

Based on our results, the decrease in the *Area* parameter was observed. This parameter represents the pool size of electron acceptors (QA) on the reducing side of PSII. The decrease of this parameter may indicate a blockage of electron transfer from the reaction centers to the quinone pool of PSII^41^. The decrease in F_m_ was also observed, which may indicate an inhibition of electron transport on the donor side of PSII, leading to an accumulation of P_680_^+^ and a decrease in the pool size of Q_A_. The decreased F_m_ may also be associated with an increase in nonphotochemical quenching. The higher ABS/RC value indicates an enlarged antenna with active and inactive reaction centers or conversion of PSII units to heat sink units along with inactive RCs^42^.

In the present study, the decrease of PF and DF curves under the influence of both heavy metals was found almost simultaneously. The I–P phase depends on the supply of electrons from PSII flowing through PSI and the transient blockade on the acceptor side of PSI^43^. Thus, it can be concluded that both metals decrease the activity of PSI^44,45^. Another possibility is that the application of heavy metals causes a decrease in the linear electron flow through both photosystems^11^.

The observed decrease in the P point confirms that the non-radiative dissipation of PSII antenna chlorophylls is increasing^44,46^ or the number of photosynthetic apparatuses with fully closed PSII RCs is decreasing^11^. These effects caused by the influence of heavy metals led to a decrease in the total PSII activity, as shown by the decrease in PI_abs_ in both cultivars.

It has been reported that DF decreases under stress conditions^47^. The curve consists of several characteristic points. The first characteristic point is the maximum (I_1_), and its decrease could indicate the photochemical accumulation of certain redox states capable of charge recombination of DF light quantum emission. These states are referred to as "light emitting". Our results showed that the I_1_ parameter was significantly affected by Ni and Cd loading in both grades. The second maximum (I_2_) indicates the non-photochemical enhancement of DF by the electrical gradient formed by PSI^48,49^. The I_2_ maximum can be detected during the decrease of the PQ pool and consequently when the disappearance of P_700_^+^ and the decrease of the transmembrane electrical gradient begin^50^. Goltsev et al.^51^ suggested that I_2_ may be associated with the dynamics of the concentration of light-emitting states of the PSII reaction center when electron transfer from Q_B_ to PQ begins rather than with the presence of the transmembrane electrical gradient. The I_2_ parameter was significantly affected by both metals, but the changes in the ‘Nira’ cultivar were detected only under the influence of 50 mg Ni, while in ‘Niga’ cultivar the changes were also detected with the other Ni treatment. In our results, the changes were observed in another characteristic point, D2. This parameter is associated with the maximum concentration of reduced P_700_. The PQ pool is reduced and the acceptor side of PSI is still inactive^51^. I_1_/I_2_ is inversely related to electron flow in PSII and has been reported to be sensitive to even mild stress^51^. Our results clearly indicate a reduced biophysical performance of the photosynthetic system in seedlings of perennial ryegrass under the influence of both metals.

The significant changes of the (I_1 _− D_2_)/D_2_ ratio can indicate that the electron transport chain efficiency was reduced as a result of the loss of PSII activity and the damage of PSI function. The I_1 _− D_2_/D_2_ parameter reflects the rate of electron transport in the PSII acceptor side, which is dependent upon the redox state of Q_A_, Q_B_, and the PQ pool^41,49^. We can confirm that the (I_1 _− D_2_)/D_2_ parameter was significantly affected by heavy metals in both cultivars.

The significant influence on the MR signal of perennial ryegrass was also observed. Schansker et al.^24^ and also Gould et al.^52^ stated that the MR signal is mainly influenced by the rate of electron flow from PSII to PSI. Moreover, the limitation of the acceptor side of PSI can be the reason for the changing MR signal^49^. Our results proved significant differences between the values of the ΔMR_fast_ and ΔMR_slow_ parameters measured in the control plants and in the plants treated by both metals. These results are well-matched with other authors, who proved that these parameters were sensitive to different stresses^45,51^ who suggested that the fast phase of the signal corresponds to the kinetics of the photoinduced changes in the P_700_ redox state. It can be significantly modified only by strong stress^24^. However, the slow phase of the MR signal, which reflects the re-reduction of P_700_^+^, could be more sensitive and decreased progressively with stress intensity^14^. Probably, the deactivation of this kinetic phase is revealed as a diminished rate of electron transport through plastoquinone to P_700_^+^, and is the reason for the more stable fast phase of the MR signal^45,51^. The decreased PSI reduction activity can also result from decreased PSII capacity to pump electrons to the intersystem electron transport chain, a disconnection between PSII and PSI), and/or the inhibition of the PSI acceptor side^46,53,54^.

It should be noted that this study showed significant differences between the tested varieties in terms of the response of the photosynthetic apparatus to stress. Significant differences between different cultivars of *Lolium perenne* seedlings to drought and salinity stress have been previously demonstrated^55,56^. Significant influence of differ heavy metals were also shown in fully developed ecotypes of this species^26^. These authors also found, that reaction of photosynthesis of this species on Cd, Pb and Zn contamination can varied among cultivars of this species. However, due to complex biological interactions, currently used methods do not always give the demanded results, so further multidirectional studies are needed.

Simultaneous measurement of PF, DF, and MR_820_ signals can be used to accurately determine electron transport through PSII and to PSI. Delayed fluorescence analysis can provide additional information on the change in antenna size heterogeneity of PSII. From this work, it appears that simultaneous measurements of PF, DF, and MR_820_ can provide broader and detailed information on structural and functional changes of PSII and PSI under heavy metal stress. This type of measurements allows to understand better the different parts of the electron transport chain.

## Materials and methods

### Plants, growth conditions and experiment design

Two turf cultivars of perennial ryegrass (*Lolium perenne* L.)—‘Niga’ and “Nira’— Małopolska Hodowla Roślin, Poland—were the objects of the experiment. Both cultivars are widely used in the Central European region, they are characterized by similar utility values and resistance to diseases. The Nira cultivar is more predisposed to less fertile soils. Previous studies have shown that this cultivar is drought and salinity resistant. The Niga cultivar has not been studied in this aspect before^54,55^. The experiment was conducted in a phytotron at the Warsaw University of Life Sciences—SGGW. Growth conditions of plants in a phytotron were set and automatically controlled during the study period. The relative humidity of air was 65%, the photoperiod for the day/night cycle was 16/8 h and radiation during the day was 95 (W m^−2^) (350 PAR) at plant height. The temperature was gradually increased (2 °C per hour) from 12 °C at night to 26 °C during the day.

The seeds of both cultivars were sown in 13 × 13 × 13 cm pots (25 pcs/pot) filled with clay sand. Pots were without holes. Five treatments of heavy metals were applied as chlorides to the substrate before sowing: 0 mg × g^−1^ of substrate (control); 50 mg Ni × g^−1^ of substrate; 100 mg Ni × 100 g^−1^ of substrate; 50 mg Cd × g^−1^ of substrate and 100 mg Cd × 100 g^−1^ of substrate. The heavy metals were applied as water solution. In addition, a single dose of the multi-component fertilizer Substral® 100 (30 g × m^−2^) was applied before sowing. All of the measurements were carried out in uniform conditions on first fully developed leaves in 6 repetitions. The measurements were made in one point in time, 8 weeks after sowing.

### The heavy metals concentration in leaves

The powdered samples were dry-mineralized in a muffle oven (Naberthern L40/11/P320) using the following time/temperature procedure: 120 °C—2 h, 200 °C–1 h, 300 °C—1 h, and 450 °C—5 h. The ashes were digested in 30% HCl (Merck suprapur) and filteredthrough a filter paper (Allen et al. 1974). The metals were determined using an atomic absorption spectrophoto-metric method with the use of the Perkin Elmer 1100 B apparatus (Perkin Elmer, 1990). Three replicate subsamples of each sample were processed. Three blanks were run with each batch of samples; thus, each sample was blank corrected. To provide a quality control (QC), the elemental content in the plant samples was determined using certified reference materials from NIST-USA (SRM 1573a—Tomato Leaves). The obtained results were in good agreement with the certified values. The recovery range was from 94 to 99%, and the accuracy was 2–4%.

### The chlorophyll and total nitrogen concentration in leaves

The concentration of chlorophylls (Chl *a*, *b* and *a* + *b*) in leaves was estimated by the spectrophotometric method. Prior to extraction, fresh samples (0.5 g) were homogenized with acetone (80%v/v), filtered thought filter paper, and made up to a final volume of 5 ml. Chlorophyll concentrations was calculated from the absorbance of the extract at 663 and 645 nm using the formula^57^ given below:

$${\text{Chl}}\,a\left( {{\text{mg}}\cdot{\text{g}}^{{ - {1}}} {\text{FW}}} \right) \, = { 11}.{75 } \times {\text{ A}}_{{{663}}} {-}{ 2}.{35 } \times {\text{ A}}_{{{645}}}$$

Chl *b* (mg·g^−1^ FW) = 18.61 × A_645 _– 3.96 × A_663_N was determined by the Kjeldahl method using a Foss Tecator (Foss Polska,).

### The chlorophyll fluorescence measurements

The M-PEA Chlorophyll Fluorescence Measurements System (Multi-Function Plant Efficiency Analyzer, Hansatech Instruments®, UK) was used. The following measurement protocol was used: measurement time—1.0 s, intensity of actinic light—3000 μmol m^−2^ s^−1^, wavelength—635 ± 10 nm. Three chlorophyll fluorescence signals were measured simultaneously: prompt chlorophyll *a* fluorescence, delayed chlorophyll *a* fluorescence, and 820 nm light reflection.

The fluorescence signal was recorded with a maximum frequency of 105 points s^–1^ (each 10 ms) within 0–0.3 ms. As a result, the collection of 118 points within 1 s was obtained. The PF is the fast kinetics of fluorescence emission from samples adapted to the dark and can be described by a fluorescence induction curve (fluorescence transient), its normalized analyze is known as the JIP-test^21^. The minimum of the curve is F_o_; it is the initial fluorescence level measured at time 0.05 ms after actinic light is applied. This point is labeled as O. The maximum fluorescence is F_m_ (labeled as point P). There are also characteristic points between O and P, labeled K (300 μs), J (2–3 ms), and I (30 ms). Chlorophyll fluorescence induction curves were obtained by the application of short pulse (in this study 1 s) of saturating light (650 nm), plotted on a logarithmic time scale is common practice to illustrate the polyphasic shape with time. The JIP-test was used to calculate parameters derived from these characteristic points of photoinduced chlorophyll fluorescence transients^21^. All parameters are described in Table 6.

**Table 6:** The description of fluorescence parameters, modified from^21^.

Area	Area above the OJIP curve
V_J_ = (F_J_ − F_o_)/(F_m_ − F_m_)	Relative variable fluorescence at the J-step
φ_Po_ = 1 − F_o_/F_m_	Maximum quantum yield of primary photochemistry (at t = 0)
φ_Eo_ = (1 − F_o_/F_m_)(1 − V_J_)	Quantum yield of electron transport (at t = 0)
φ_Ro_ = (1 − F_o_/F_m_)(1 − V_I_)	Quantum yield of reduction of end electron acceptors at the PSI acceptor side (RE)
φ_Do_ = F_o_/F_m_	Quantum yield (at t = 0) of energy dissipation
Ψ_Eo_ = 1 − V_J_	Probability (at t = 0) that a trapped exciton moves an electron into the electron transport chain beyond Q_A_^–^
δ_Ro_ = (1 − V_I_)/(1 − V_J_)	Efficiency/probability with which an electron from the intersystem electron carriers moves to reduce end electron acceptors at the PSI acceptor side (RE)
t(F_m_)	Time (in m/s) to reach the maximal fluorescence intensity F_m_
N	The number of rotation, amount of reduced Q_A_ in time from 0 to t(F_m_)
PI_abs_ = γ_RC_/(1 − γ_RC_) × φ_Po_/(1 − φ_Po_) × Ψ_Eo_/(1 − Ψ_Eo_)	Performance index (potential) for energy conservation from exciton to the reduction of intersystem electron acceptors
PI_total_ = PI_ABS_ × δ_Ro_/(1 − δ_Ro_)	Performance index (potential) for energy conservation from exciton to the reduction of PSI end acceptors
ABS/RC = (1 − γ_RC_)/γ_RC_	Absorption flux (of antenna Chls) per RC
TR_o_/RC = M_o_(1/V_J_)	Trapping flux (leading to Q_A_ reduction) per RC
ET_o_/RC = M_o_(1/V_J_)Ψ_o_	Electron transport flux (further than Q_A_^–^) per RC
RE_o_/RC = M_o_(1/V_J_)(1 − V_I_)	Electron flux reducing end electron acceptors at the PSI acceptor side per RC
DI_o_/RC = (ABS/RC − TR_o_/RC)	Dissipated energy flux per RC (at t = 0)
RC/CS_o_ = φ_Po_ (V_J_/M_o_) F_o_	Density of RCs (Q_A_ reducing PSII reaction centres)
I_1_ and I_2_	Maxima of DF induction curve
D_2_	Minimum of DF induction curve
MR_o_	Modulated 820 nm reflection intensity at time “0”
MR_min_	Minimum of modulated 820 nm reflection intensity
MR_max_	Maximum of modulated 820 nm reflection intensity
ΔMR_fast_	Fast phase (oxidation) of reflection intensity = MR_o_ − MR_min_
ΔMR_slow_	Slow phase (reduction) of reflection intensity = MR_max_ − MR_min_

To better visualize the influence of the two heavy metals on the dynamics of the chlorophyll transients, the relative variable fluorescence intensity (V_t_) was calculated. As the next stage, the differences of relative variable fluorescence intensity (ΔV_t_) were calculated by subtracting the normalized fluorescence values (between O and P steps) recorded in control plants and under stress. V_t_ and ΔV_t_ were calculated according to the formulas:

1 $${\text{V}}_{{\text{t}}} = \, \left( {{\text{F}}_{{\text{t}}} {-}{\text{ F}}_{{\text{o}}} } \right)/ \, \left( {{\text{F}}_{{\text{m}}} {-}{\text{F}}_{{\text{o}}} } \right)$$

2 $$\Delta {\text{V}}_{{\text{t}}} ,_{{{\text{stress}}}} = {\text{ V}}_{{{\text{t}},{\text{stress}}}} {-}{\text{V}}_{{{\text{t}},{\text{control}}}}$$

The characteristic points (I_1_, I_2_ and D_2_) of DF curves were assessed according to Goltsev et al.^23^. The I_1_ point is the first maximum of the curve, the I_2_ point is second maximum, and D_2_ is second minimum of curve. Moreover, two ratios were calculated to better illustrate the influence of metals on DF induction curves: (I_1 _− D_2_)/D_2_ and I_1_/I_2_^23^. However, our obtained data did allow us to identify D_1_ point.

The MR signal was measured simultaneous as PF and DF. The ΔMR_fast_ and ΔMR_slow_ parameters were calculated.

### Growth parameters

The growth parameters of *L. perenne* cultivars were determined 14 days after sowing. The length of shoots and the length of roots were determined. These features were determined on five randomly chosen seedlings of the tested cultivars taken from each pot (in total 5 seedlings × 6 repetitions). The WinRhizo PRO 2009 (Regent Instruments Inc., Canada) scanned image technique was used for the analysis of plant materials.

### Statistical analysis

All ChlF parameters were statistically analyzed using the one-way test ANOVA. REGWQ test was used as a post-hoc test with a confidence level of 0.05. The relationships between the studied characteristics and their changes under heavy metal conditions were determined based on principal component analysis (PCA) and by correlation analysis. Statistica 13.0 program (TIBCO Software Inc., USA) and IBM SPSS Statistics ver. 28 were used to perform the statistical analysis.

## Conclusions

To the best of our knowledge, the analysis of simultaneous measurements of PF, DF, and MR_820_ signals was performed for the first time with the seedlings of perennial ryegrass grown under heavy metal stress. Although the response of the photosynthetic machinery to this stress is a very complicated process, this analysis proved that the comprehensive analyses of chlorophyll *a* fluorescence signals are a very accurate tools to diagnose the influence of this stress on photosynthetic process. Our results revealed that both metals decreased the activity of PSII and PSI. This may be due to an increase in nonradiative dissipation of PSII antenna chlorophylls, a decrease in PSII antenna size or/and a decrease in the number of photosynthetic apparatuses with fully closed PSII RCs. Based on DF analysis, we concluded that both metals could lead to photochemical accumulation of certain redox states as well as a decrease in the PQ pool. In addition, our results evidently showed that the efficiency of electron transport chain was reduced by the loss of PSII activity and damage to the function of PSI. A significant effect on the MR signal of perennial ryegrass was also observed. This may indicate the limitation of the electron flow rate from PSII to PSI or of the acceptor side of PSI. The decreased PSI reduction activity may also result from decreased PSII capacity to pump electrons into the cross-system electron transport chain, disruption of the connection between PSII and PSI, and/or inhibition of the PSI acceptor site. Significant differences were demonstrated between the perennial ryegrass cultivars tested with respect to their responses to both heavy metal stresses. These findings may lead to the selection of plants with a higher potential for photosynthetic performance under contaminated soil conditions that can be successfully applied to adjacent roads or other post-industrial sites to be reclaimed. In order to more precisely determine the impact of individual heavy metals, when planning subsequent experiments, it is proposed to extend the scope of research with a larger number of concentrations.

## Data Availability

The datasets used and analyzed during the current study available from the corresponding author on reasonable request.

## Abbreviations

ChFl: Chlorophyll *a* fluorescence
PSII and PSI: Photosystem I and II
ABS: Energy fluxes of absorption of light energy
TR: Trapping of excitation energy
ET: Electron transport (ET)
RC: Reaction center
CS: Measured area of the samples
PF: Prompt fluorescence
DF: Delayed fluorescence
MR_820_: Modulated reflectance signal at 820 nm
PQ: Plastoquinone
PC: Plastocyanin
Q_A_ and Q_B_: Primary and secondary electron acceptor

## References

[1] Jamali MK, Kazi TG, Arain MB, Afridi HI, Jalbani N, Memon AR (2008). Heavy metal contents of vegetables grown in soil, irrigated with mixtures of wastewater and sewage sludge in Pakistan, using ultrasonic-assisted pseudo-digestion. J. Agron. Crop Sci..

[2] Lombi E, Zhao FJ, Dunham SJ, McFrath SP (2000). Cadmium accumulation in populations of *Thlaspi caerulescens* and *Thlaspi goesingense*. New Phytol..

[3] Radziemska M, Mazur Z, Jeznach J (2014). Effect of zeolite and halloysite on accumulation of trace element san maize (*Zea mays* L.) in nickel contaminated soils. Fresenius Environ. Bull..

[4] Radziemska M, Gusiatin ZM, Cydzik-Kwiatkowska A, Cerdàc A, Pecinade V, Bęś A, Dattad R, Majewski G, Mazur Z, Dzięcioł J, Danish S, Brtnický M (2021). Insight into metal immobilization and microbial community structure in soil from a steel disposal dump phytostabilized with composted, pyrolyzed or gasified wastes. Chemosphere.

[5] Dąbrowski P, Poniecka B, Baczewska AH, Gworek B (2016). Wpływ transportu drogowego na zanieczyszczenie gleb i roślin ołowiem i chromem. Przemysł chemiczny.

[6] Gworek B, Dąbrowski P, Poniecka B, Wrzosek J (2011). Wpływ ruchu drogowego na zanieczyszczenie gleb i roślin rtęcią. Przemysł chemiczny.

[7] Athar R, Masood A (2002). Heavy metal toxicity; effect on plant growth and metal uptake by wheat, and on free living Azotobacter. Water Air Soil Pollut..

[8] Munzuroglu Ö, Gekil H (2002). Effects of metals on seed germination, root elongation and coleoptile and hypocotyl growth in *Triticum aestivum* and *Cucumis sativus*. Arch. Environ. Contam. Toxicol..

[9] Fusconi A, Gallo C, Camusso W (2007). Effects of cadmium on root apical meristems of *Pisum sativum* L.: Cell viability, cell proliferation and microtubule pattern as suitable markers for assessment of stress pollution. Mutat. Res..

[10] Kalaji HM, Jajoo A, Oukarroum A, Brestic M, Zivcak M, Samborska IA, Cetner MD, Łukasik I, Goltsev V, Ladle RJ (2016). Chlorophyll a fluorescence as a tool to monitor physiological status of plants under abiotic stress conditions. Acta Physiol. Plant..

[11] Paunov M, Koleva L, Vassilev A, Vangronsveld J, Goltsev V (2018). Effects of different metals on photosynthesis: Cadmium and zinc affect chlorophyll fluorescence in durum wheat. Int. J. Mol. Sci..

[12] Jahan MS, Guo S, Baloch AR, Sun J, Shu S, Wang Y, Ahammed GJ, Kabir K, Roy R (2020). Melatonin alleviates nickel phytotoxicity by improving photosynthesis, secondary metabolism and oxidative stress tolerance in tomato seedlings. Ecotox. Environ. Saf..

[13] Aqeel M, Khalid N, Tufail A, Ahmad RZ, Akhter MS, Luqman M, Javed MT, Irshad MK, Alamri S, Hashem M, Noman A (2021). Elucidating the distinct interactive impact of cadmium and nickel on growth, photosynthesis, met-al-homeostasis, and yield responses of mung bean (*Vigna radiata* L.) cultivars. Environ. Sci. Pollut. Res..

[14] Dąbrowski P, Baczewska-Dąbrowska AH, Bussotti F, Pollastrini M, Piekut K, Kowalik W, Wróbel J, Kalaji HM (2021). Photosynthetic efficiency of *Microcystis* ssp. under salt stress. Environ. Exp. Bot..

[15] Kalaji HM, Rastogi A, Živčák M, Brestic M, Daszkowska-Golec A, Sitko K, Alsharafa KY, Lotfi R, Stypiński P, Samborska IA, Cetner MD (2018). Prompt chlorophyll fluorescence as a tool for crop phenotyping: An example of barley landraces exposed to various abiotic stress factors. Photosynthetica.

[16] Kalaji HM, Bosa K, Kościelniak J, Żuk-Gołaszewska K (2011). Effects of salt stress on photosystem II efficiency and CO_2_ assimilation of two Syrian barley landraces. Environ. Exp. Bot..

[17] Stirbet A (2011). On the relation between the Kautsky effect (chlorophyll *a* fluorescence induction) and photosystem II: Basics and applications of the OJIP fluorescence transient. J. Photochem. Photobiol. B Biol..

[18] Oukarroum A, Madidi SE, Schansker G, Strasser RJ (2007). Probing the responses of barley cultivars (*Hordeum vulgare* L.) by chlorophyll a fluorescence OLKJIP under drought stress and re-watering. Eviron. Exp. Bot..

[19] Meng LL, Song JF, Wen J, Zhang J, Wei JH (2016). Effects of drought stress on fluorescence characteristics of photo-system II in leaves of *Plectranthus scutellarioides*. Photosynthetica.

[20] Strasser, B., Strasser, R. Measuring fast fluorescence transients to address environmental questions: the JIP-test. In: Garab, G. (Ed.), Photosynthesis: From Light to Biosphere 1995, 5, 977–980.

[21] Strasser RJ, Tsimilli-Michael M, Qiang S, Goltsev V (2010). Simultaneous in vivo recording of prompt and delayed fluores-cence and 820-nm reflection changes during drying and after rehydration of the resurrection plant *Haberlea rhodopensis*. Biochim. Biophys. Acta Bioenerg..

[22] Goltsev V, Zaharieva I, Chernev P, Kouzmanova M, Kalaji HM, Yordanov I, Krasteva V, Alexandrov V, Stefanov D, Allakhverdiev SI, Strasser RJ (2012). Drought-induced modifications of photosynthetic electron transport in intact leaves: analysis and use of neural networks as a tool for a rapid non-invasive estimation. Biochim. Biophys. Acta Bioenerg..

[23] Goltsev V, Zaharieva I, Chernev P, Strasser RJ (2009). Delayed fluorescence in photosynthesis. Photosynth. Res..

[24] Schansker G, Srivastava A, Strasser RJ (2003). Characterization of the 820-nm transmission signal paralleling the chlorophyll *a* fluorescence rise (OJIP) in pea leaves. Funct. Plant Biol..

[25] Dąbrowski P, Pawluśkiewicz B, Kalaji HM, Baczewska AH (2013). The effect of light availability on leaf area index, biomass production and plant species composition of park grasslands in Warsaw. Plant Soil Environ..

[26] Żurek G, Wiewiórka B, Rybka K, Prokopiuk K (2022). Different response of perennial ryegrasss–Epichloë endophyte symbiota to the elevated concentration of heavy metals in soil. J. Appl. Gen..

[27] Xu B, Li F, Shan L, Ma Y, Ichizen N, Huang J (2006). Gas exchange, biomass partition, and water relationships of three grass seedlings under water stress. Weed Biol. Manag..

[28] Faseela P, Sinisha AK, Brestič M, Puthur JT (2020). Chlorophyll *a* fluorescence parameters as indicators of a particular abiotic stress in rice. Photosynthetica.

[29] Bussotti F, Gerosa G, Digrado A, Pollastrini M (2020). Selection of chlorophyll fluorescence parameters as indicators of photosynthetic efficiency in large scale plant ecological studies. Ecol. Indicators.

[30] Kalaji HM, Schansker G, Ladle RJ, Goltsev V, Bosa K, Allakhverdiev SI (2014). Frequently asked questions about in vivo chlorophyll fluorescence: Practical issues. Photosynth. Res..

[31] Suresh Kumar K, Dahms H-U, Lee J-S, Kim HC, Lee WC, Shin K-H (2014). Algal photosynthetic responses to toxic metals and herbicides assessed by chlorophyll *a* fluorescence. Ecotoxicol. Environ. Saf..

[32] Goltsev VN, Kalaji HM, Paunov M (2016). Variable chlorophyll fluorescence and its use for assessing physiological condition of plant photosynthetic apparatus. Russ. J. Plant. Physiol..

[33] Chen Q, Zhang X, Liu Y, Wei J, Shen W, Shen Z, Cui J (2017). Heminmediated alleviation of zinc, lead and chromium toxicity is associated with elevated photosynthesis, antioxidative capacity; suppressed metal uptake and oxidative stress in rice seedlings. Plant Growth Regul..

[34] Yadav S (2010). Heavy metals toxicity in plants: an overview on the role of glutathione and phytochelatins in heavy metal stress tolerance of plants. S. Afr. J. Bot..

[35] Khalid N, Noman A, Sanaullah T, Akram MA, Aqeel M (2018). Vehicle pollution toxicity induced changes in physiology, defence system and biochemical characteristics of *Calotropis procera* L. Chem. Ecol..

[36] Li Q, Lu Y, Shi Y, Wang T, Ni K, Xu L, Liu S, Wang L, Xiong Q, Giesy JP (2013). Combined effects of cadmium and fluoranthene on germination, growth and photosynthesis of soybean seedlings. J. Environ. Sci..

[37] Shah SS, Mohammad F, Shafi M, Bakht J, Zhou W (2011). Effects of cadmium and salinity on growth and photosynthesis parameters of Brassica species. Pak. J. Bot..

[38] Figlioli F, Sorrentino MC, Memoli V, Arena C, Maisto G, Giordano S, Capozzi F, Spagnuolo V (2019). Overall plant responses to Cd and Pb metal stress in maize: Growth pattern, ultrastructure, and photosynthetic activity. Environ. Sci. Pollut. Res..

[39] Ci D, Jiang D, Wollenweber B, Dai T, Jing Q, Cao W (2010). Cadmium stress in wheat seedlings: Growth, cadmium accumulation and photosynthesis. Acta Physiol. Plant..

[40] Younis U, Qayyum MF, Shah MHR, Danish S, Shahzad AN, Malik SA, Mahmood S (2015). Growth, survival, and heavy metal (Cd and Ni) uptake of spinach (*Spinacia oleracea*) and fenugreek (*Trigonella corniculata*) in a biochar-amended sewage-irrigated contaminated soil. J. Plant Nutr. Soil. Sci..

[41] Mehta P, Jajoo A, Mathur S (2010). Chlorophyll *a* fluorescence study revealing effects of high salt stress on photosystem II in wheat leaves. Plant Physiol. Biochem..

[42] Mathur S, Mehta P, Jajoo A (2013). Effects of dual stress (high salt and high temperature) on the photochemical efficiency of wheat leaves (*Triticum aestivum*). Physiol. Mol. Biol. Plant..

[43] Schansker G, Tóth SZ, Strasser RJ (2005). Methylviologen and dibromothymoquinone treatments of pea leaves reveal the role of photosystem I in the Chl *a* fluorescence rise OJIP. BBA-Bioenergetics.

[44] Oukarroum A, Bussotti F, Goltsev V, Kalaji HM (2015). Correlation between reactive oxygen species production and photo-chemistry of photosystems I and II in *Lemna gibba* L. plants under salt stress. Environ. Exp. Bot..

[45] Salvatori E, Fusaro L, Strasser RJ (2015). Effects of acute O3 stress on PSII and PSI photochemistry of sensitive and re-sistant snap bean genotypes (*Phaseolus vulgaris* L.), probed by prompt chlorophyll “a” fluorescence and 820 nm modulated reflectance. Plant Physiol. Biochem..

[46] Oukarroum A., Gharous M.E., Goltsev V. Desiccation-induced changes of photosynthetic transport in Parmelina tili-acea, (Hoffm.) Ach. Analysed by simultaneous measurements of the kinetics of prompt fluorescence, delayed fluorescence and modulated 820 nm reflection. *J. Lumin.* (2018).

[47] Zhang L, Xing D (2008). Rapid determination of the damage to photosynthesis caused by salt and osmotic stresses using delayed fluorescence of chloroplasts. Photochem. Photobiol. Sci..

[48] Goltsev V, Chernev P, Zaharieva I, Lambrev P, Strasser R (2005). Kinetics of delayed chlorophyll a fluorescence registered in milliseconds time range. Photosynth. Res..

[49] Lazár D (2009). Modelling of light-induced chlorophyll a fluorescence rise (O–J–I–P transient) and changes in 820 nm-transmittance signal of photosynthesis. Photosynthetica.

[50] Zaharieva I, Goltsev V (2003). Advances on Photosystem II investigation by measurement of delayed chlorophyll fluorescence by a phosphoroscopic method. Photochem. Photobiol..

[51] Goltsev V, Zaharieva I, Chernev P, Kouzmanova M, Kalaji HM, Yordanov I, Krasteva V, Alexandrov V, Stefanov D, Allakhverdiev SI, Strasser RJ (2012). Drought-induced modifications of photosynthetic electron transport in intact leaves: analysis and use of neural networks as a tool for a rapid non-invasive estimation. BBA-Bioenergetics.

[52] Gould PD, Diaz P, Hogben C, Kusakina J, Salem R, Hartwell J, Hall A (2009). Delayed fluorescence as a universal tool for the measurement of circadian rhythms in higher plants. Plant J..

[53] Kan X, Ren JJ, Chen TT, Cui M, Zhou R, Zhang Y, Liu H, Deng D, Yin Z (2017). Effects of salinity on photosynthesis in maize probed by prompt fluorescence, delayed fluorescence and P700 signals. Environ. Exp. Bot..

[54] Zhou R, Kan X, Chen J, Hua H, Li Y, Ren J, Feng K, Liu H, Deng D, Yin Z (2019). Drought-induced changes in pho-tosynthetic electron transport in maize probed by prompt fluorescence, delayed fluorescence, P700 and cyclic electron flow signals. Environ. Exp. Bot..

[55] Dąbrowski P, Kalaji MH, Baczewska AH, Pawluśkiewicz B, Mastalerczuk G, Borawska-Jarmułowicz B, Paunov M, Goltsev V (2017). Delayed chlorophyll a fluorescence, MR 820, and gas exchange changes in perennial ryegrass under salt stress. J. Lumin..

[56] Dąbrowski P, Baczewska-Dąbrowska AH, Kalaji HM, Goltsev V, Paunov M, Rapacz M, Wójcik-Jagła M, Pawluśkiewicz B, Bąba W, Brestic M (2019). Exploration of chlorophyll a fluorescence and plant gas exchange parameters as indicators of drought tolerance in perennial ryegrass. Sensors.

[57] Lichtenthaler HK (1987). Chlorophylls and carotenoids, the pigments of photosynthetic biomembranes. Methods Enzymol..

